# The increasing burden of the 2-week wait colorectal cancer pathway in a single centre: the impact of faecal immunochemical tests

**DOI:** 10.1308/rcsann.2022.0138

**Published:** 2023-01-23

**Authors:** N Farkas, JW O’Brien, L Palyvos, W Maclean, S Benton, T Rockall, I Jourdan

**Affiliations:** Royal Surrey NHS Foundation Trust, UK

**Keywords:** Two-week pathway, Colonoscopy, Colorectal cancer, Endoscopy

## Abstract

**Introduction:**

Two-week wait (TWW) volume and colorectal cancer (CRC) detection pose an increasing challenge for NHS cancer services. Primary aims were to assess the introduction of faecal immunochemical tests (FIT) into clinical practice at our centre, the impact on TWW referral volume and CRC diagnoses, and to provide an update to previously published work. A secondary aim was to correlate FIT value and investigation.

**Methods:**

TWW CRC data following incorporation of FIT into clinical practice were analysed (1 June 2019–31 July 2021). Parameters assessed were monthly referral volume, CRC detection, primary care FIT volume and secondary care investigations. Referrals and CRC detection rates were compared with previously published data (2009–2019). Data relating to primary care FIT were collated from Berkshire and Surrey Pathology Services.

**Results:**

TWW referrals increased 360% (2009–2020). CRC incidence decreased from 8.87% to 3.24%. Following incorporation into clinical practice, primary care FIT requests have increased to >450/month and accompanied 1,722/4,796 referrals. CRC incidence is static (3–4%). Patients with FIT <10µg Hb/g faeces undergo radiological imaging more commonly, whereas FIT-positive patients are more likely to undergo endoscopy, although the difference is not statistically significant.

**Conclusions:**

No significant change in CRC diagnosis was observed, despite increasing TWW referrals. Increasing utilisation of FIT in both primary and secondary care has helped maintain CRC detection while avoiding diagnostic delay. This study supports growing evidence highlighting the value of FIT in triage, referral and TWW investigation. FIT appears increasingly important for allocating secondary care resources (endoscopy), while guiding primary care referral. Additional low-cost strategies to determine prioritisation or reassurance (e.g. repeat FIT) require further evaluation.

## Introduction

Two-week wait (TWW) referral volume and subsequent rate of colorectal cancer (CRC) detection pose an increasing challenge for NHS cancer services. In a recent review on the impact of fast-track referrals and CRC outcomes in the UK, Thompson *et al* concluded, ‘referrals are overwhelming hospital resources without producing the expected increases in survival’.^1^ Similarly, Jones *et al* concluded that changes to the TWW referral pathway in 2015 have ‘led to an increased number of patients being referred, but have not resulted in a change in the rate of colorectal cancer detection’.^2^

National Institute for Health and Care Excellence (NICE) guidelines recommend TWW pathways utilise prompt triage of primary care referrals (within 14 days) before patients are seen in a face-to-face or telephone clinic, whereupon secondary investigations such as endoscopy and computerised tomography (CT) are employed.^3^ Some services utilise ‘straight to test’ from receipt of the referral.

Over the preceding 12 years, TWW referral pathways have been amended on a number of occasions, resulting in expanded referral criteria for suspected CRC patients. Such changes include the amended NICE guidance in 2015 (NG12) where ‘any change in bowel habit’ met the new referral criteria,^3^ and the DG30 update in 2017 to adopt quantitative faecal immunochemical testing (FIT) for low-risk patients.^4^ Similarly, the impact of the coronavirus disease 2019 (COVID-19) pandemic has affected the provision of healthcare globally, with almost all areas of the NHS placed under additional strain.^5^ The unprecedented nature of this has challenged the ability of the NHS to deliver high-quality care for suspected cancer patients.^6^

The introduction of quantitative FIT has proved a useful clinical adjunct to clinicians in both primary and secondary care when investigating symptomatic patients.^7^ However, much of the evidence base surrounding FIT has been established in the bowel cancer screening setting. Consequently, there is a relative scarcity of pertinent literature in comparison. The pandemic has seen an accelerated incorporation of FIT into many management pathways for suspected CRC patients.^8,9^ However, limited data exist regarding the impact of FIT on primary care referral volume and on clinical decision-making in secondary care.

The primary aims of this paper were to assess the introduction of FIT into clinical practice, the impact on TWW referral numbers and CRC diagnoses in this centre over the past 2 years and to provide an update to previously published work.^10^ Secondary aims were to evaluate the impact of FIT on utilisation of diagnostic tests and investigations.

## Methods

The Royal Surrey NHS Foundation Trust is a district general hospital in England providing cancer services for 1.3 million patients. We extracted data from a prospectively maintained database for all TWW CRC referrals from 1 June 2019 to 31 July 2021. The measures assessed were monthly referral volume, CRC detection, FIT requested in primary care and choice of secondary care investigation. Referral volume and CRC detection rates were compared with data from 1 January 2009 to 30 June 2019. This timeframe was chosen to enable outcomes to be compared before and after the introduction of FIT into clinical practice.

Data relating to all primary care FIT requests were collated from Berkshire and Surrey Pathology Services (BSPS). Requests related only to those general practitioner (GP) practices that referred to our centre. Data were included if a FIT request was made before, or at the time of, onward referral to secondary care.

FIT data were categorised into <10µg Hb/g faeces, 10–100µg Hb/g faeces and >100µg Hb/g faeces. The relationship between FIT group and secondary care investigations required quarterly evaluation to allow for sufficient numbers.

The paper was written in line with STROBE guidelines.^11^ Statistical analysis was conducted where appropriate. As this is an observational cohort study, outcomes have been reported objectively to minimise outcome-reporting bias. Two authors reviewed the data independently to help reduce confirmation bias when interpreting results.

## Results

Table 1 demonstrates a year-on-year increase in the number of referrals on the TWW pathway, until 2019. Following the introduction of FIT in 2019, TWW referral volume has remained relatively stable. The incidence of CRC detected among TWW referrals has reduced from 8.87% (2009) to 3.24% (2020) over this period, while CRC rates have remained static throughout the timeframe assessed. The number of referrals per CRC diagnosis has increased by a factor of 3.6. From 2016 onwards (following amendments to NICE guidance) larger referral volumes are noted, with resulting increases in the ratio of referrals to cancer diagnosis. When analysed by month, there was a linear increase in referral volume (not shown in Table 1). As expected from the static yearly CRC incidence, there was minimal variation in monthly cancers detected. A single reduction in referrals was observed in April 2020, correlating with the first wave of the COVID-19 pandemic.

**Table 1:** Number of TWW referrals and CRC cancer diagnoses

**Year**	**No. TWW referrals**	**No. CRC** **cancers**	**Annual incidence of CRC among TWW referrals (%)**	**Ratio of TWW referrals: CRC**
2009	564	50	8.87	11.3:1
2010	552	64	11.59	8.6:1
2011	578	48	8.30	12.0:1
2012	816	54	6.62	15.1:1
2013	734	52	7.08	14.1:1
2014	1008	51	5.06	19.8:1
2015	1232	71	5.76	17.4:1
2016	1296	43	3.32	30.1:1
2017	1371	56	4.08	24.5:1
2018	1707	72	4.22	23.7:1
2019	2139	86	4.02	24.9:1
2020	2035	66	3.24	30.8:1
2021 (Jan–Jul)	1412	50	3.54	28.2:1

CRC = colorectal cancer; TWW = two-week wait

**Figure 1:**
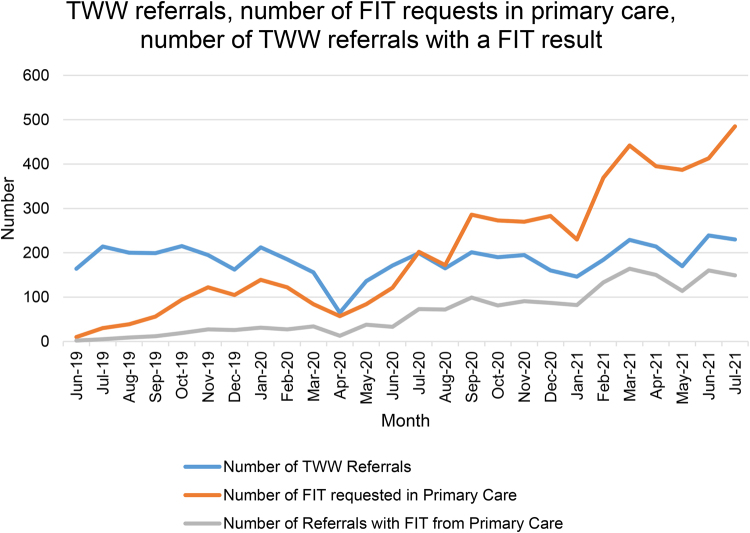
Number of TWW referrals and CRC cancer diagnoses. CRC = colorectal cancer; TWW = two-week wait

**Figure 2:**
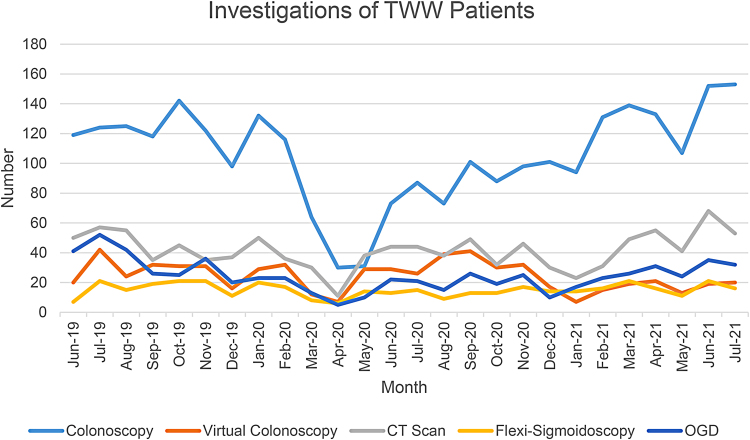
TWW investigations per month. CT = computed tomography; OGD = oesophago-gastro duodenoscopy; TWW = two-week wait

Table 2 demonstrates three timeframes over 12 years: pre and post-NG12 guidance, and following incorporation of FIT into clinical practice. There were 4,796 patients referred during the period studied. The mean number of TWW referrals per month was 191.8 (SD 36.0). Over this timeframe, 169 CRC were diagnosed. The mean number of CRCs detected per month was 6.8 (SD 2.5). On average, for each CRC diagnosed, 28.4 referrals are seen.

**Table 2:** Comparison of pre-NG12 guidance, post-NG12 and incorporation of FIT into clinical practice

** **	**Timeframe**	**Total TWW referrals**	**Mean monthly TWW referrals**	**CRC diagnosis**	**Mean monthly CRC detection rate**	**Ratio of TWW referrals: CRC**
Pre-NG12 guidance change	2009 (January) – 2015 (June)	4,805	61.6	352	4.5	13.7:1
Post-NG12 guidance change	2015 (July) – 2019 (May)	5,843	127.0	242	5.3	24.1:1
Incorporation of FIT into clinical practice	2019 (June) – 2021 (July)	4,796	191.8	169	6.8	28.4:1

CRC = colorectal cancer; FIT = faecal immunochemical testing; NG12 = amended NICE guidance in 2015; NICE = National Institute for Health and Care Excellence; TWW = two-week wait

Figure 1 demonstrates the increasing utilisation of FIT in primary care compared with TWW referral volume. Of the 4,796 TWW referrals, 1,722 were accompanied by a FIT result sent before, or at the time of, referral from primary care.

Table 3 demonstrates CRC by FIT value. Most CRC were detected in the positive FIT groups. The largest volume of CRC was in the >100μg Hb/g group, with 6.7 referrals per cancer diagnosis. In the FIT negative (<10 μg Hb/g) and 10–100 μg Hb/g groups, there were 795 and 32.4 referrals per cancer, respectively.

**Table 3:** TWW referral volume, FIT value and CRC

**FIT value (μg Hb/g faeces)**	**Number of TWW referrals**	**Percentage of TWW referrals (%)**	**Number of CRC detected**	**Percentage of CRC with FIT (%)**	**Ratio of TWW referrals: CRC**
<10	795	16.6	1	1.7	795:1
10–100	679	14.2	22	36.6	32.4:1
>100	248	5.2	37	61.7	6.7:1
No FIT	3,074	64.1	109	–	35.5:1
Total	4,796	–	169	–	–

CRC = colorectal cancer; FIT = faecal immunochemical testing TWW = two-week wait

Figure 2 highlights the investigations utilised for TWW patients during the timeframe assessed. When analysed by month (as a proportion of all investigations) CT and virtual colonoscopy increased transiently during the first wave of the pandemic. Conversely, there was a proportional drop in colonoscopy utilisation. This subsequently returned to prepandemic volume by December 2020.

Analysis of secondary care TWW investigation based on FIT status was undertaken per quarter. Subgroups assessed were FIT <10, 10–100, >100μg Hb/g and none (FIT not performed). For patients with FIT, subtle varying trends were noted across the quarters; the incorporation of FIT into the pathway resulted in a slightly higher proportion of colonoscopy being undertaken in patients with a positive result. Proportionally, type of investigation undertaken in the FIT >100μg Hb/g subgroup did not differ significantly to the 10–100μg Hb/g subgroup. In referrals without FIT (3,074 patients), choice of investigation has remained relatively constant. Patients with negative FIT undergo less colonoscopy and virtual colonoscopy and a higher proportion of CT scans (when compared with FIT values of 10–100 and >100μg Hb/g).

## Discussion

The volume of TWW referrals significantly increased over 12 years with nearly four times as many TWW referrals in 2020 (2,035) and 2019 (2,139) compared with 2009 (564) (Table 1). This is an increase on previously published data by our centre from 2009 to 2014, which showed a mean of 709 TWW referrals annually.^10^ There were 1,414 TWW referrals between April 2017 and April 2018, with 62 cancers detected. In comparison, there were 2,139 TWW referrals were made in 2019, with 86 cancers detected.

Interpretation of data should be contextualised by time period; pre- and postintroduction of the NG12 guideline in 2015, and following incorporation of FIT into clinical practice in 2019 (Table 2). The NICE guidance in 2015 lowered the referral threshold to reduce diagnostic delay and identify cancer at an earlier stage. Well-documented challenges include widening of the referral net and an associated reduction in the positive predictive value of the pathway.^1,12^ However, the symptom profile of patients referred with suspected CRC is widely reported and contributed to the referral criteria.^3^

Figure 1 demonstrates how GPs have embraced FIT following its introduction in 2019, despite a lack of national FIT referral guidance until very recently (locally, it was only a recommendation that FIT accompany urgent referral). GPs appear to be using it as an adjunct to guide decision-making in primary care, with more than twice as many FIT requests compared with TWW referrals by July 2021. Evidently, not all FIT requests result in onward referral and this cohort of nonreferred patients warrants additional study. This finding supports a survey by the Association of Coloproctology of Great Britain and Ireland (ACPGBI); 62.5% of members think FIT has helped limit referral volume.^8^ However, it cannot be definitively concluded that the plateauing of referrals is a direct consequence of FIT; a natural levelling should be expected with fixed referral criteria for a stable local population.

The ratio of referrals to cancer increased from 11.3 (2011) to 28.2 (2021) following guideline changes and incorporation of FIT. Of the 60 cancers detected (from the 1,722 referrals with FIT), one was in a patient with a negative FIT (<10µg Hb/g faeces) (Table 3). In contrast, 14.4% of referrals with FIT >100µg Hb/g faeces accounted for 61.7% of CRC detected. These findings are in keeping with larger studies that confirm the high sensitivity and specificity of FIT.^13^

TWW investigations are highlighted in Figure 2. The period of UK government lockdown (March–May 2020) had a considerable impact on clinical practice, with preference towards nonaerosol-generating investigations such as CT and virtual colonoscopy. Reduction in TWW referrals and endoscopy was observed, with numbers rebounding in subsequent months, and colonoscopy has since increased to above prepandemic levels (Figure 2). Despite this fluctuation, there was no reduction in monthly CRC detection rates, suggesting that cancer diagnoses were not missed at this centre. Conversely, the large English population study by Morris *et al*, reporting on impact on CRC following the first wave of the pandemic, concluded that at least 3,500 fewer CRCs were diagnosed compared with 2019.^14^

The rapid roll out of FIT and telephone triage may explain maintenance of the prepandemic CRC detection rate at this centre, despite reduced access to investigations.^15^ Modifying the TWW pathway to identify patients with a positive FIT and high-risk symptoms allowed prioritisation for endoscopy. To mitigate risk, our centre employed the use of a patient tracker as a safety net for those not triaged to investigation immediately; only one patient with negative FIT went on to be diagnosed with CRC. The benefits of rationing endoscopic provision are further supported by Loveday *et al*, who describe the use of strategies to mitigate pandemic-related delays. Prioritisation of symptomatic referrals with FIT >10mcg/g Hb faeces would avoid 89% of avoidable deaths while reducing the requirement for colonoscopy by >80%.^16^

It is evident from this dataset and previously published work that our centre is seeing increasing referral numbers and demand on endoscopy. This is in keeping with the 15% increase in gastrointestinal investigations observed nationally.^10,17^ If demand continues on this trajectory, a tipping point may be reached whereby capacity is overwhelmed. Rationing of endoscopy and other services will result in delayed or missed CRC diagnoses; therefore, strategies and adjuncts are required to address this. FIT status already appears to influence secondary care decision-making, but there remains potential for additional utilisation—a third of urgent TWW colonoscopy is being performed on patients without a FIT sample, and there clearly remains significant scope to refine the pathway further.

The British Society of Gastroenterology (BSG) and ACPGBI have recently released a number of recommendations including a referral flow chart.^18^ With regard to safety netting, the guidance states ‘some patients with symptoms of suspected colorectal cancer may be managed in primary care if fHb <10μg Hb/g, and appropriate safety netting is in place’. Those with negative FIT, but ongoing clinical concern and persistent/unexplained symptoms should be referred to secondary care. Targeted investigation of symptomatic patients, in a cost-effective manner, can be facilitated from primary care. This could also reduce the risk of missed CRC. The impact of such guidance is likely to have a significant effect enabling supported decision-making in primary care. This will help refine the triage of suspected CRC patients and ultimately reduce the burden on the TWW pathway and endoscopy.

Strategies incorporating FIT with safety netting may enhance investigation on the TWW pathway. The utilisation of ‘straight to test’ colonoscopy is such an example and has been shown to be cost-effective, feasible and reduces time to diagnosis.^19,20^ However, this approach does not necessarily lessen the burden on endoscopy. Increasing the proportion of referrals that have a FIT result may enhance prioritisation of this limited resource, especially for use in those patients with a positive FIT and highest risk of cancer. This group could be triaged straight to colonoscopy or virtual colonoscopy. Both our own data and larger studies confirm that a negative FIT significantly reduces the likelihood of CRC, and therefore such patients could be downgraded from the urgent pathway and triaged to radiology or sigmoidoscopy. Recent evidence demonstrates FIT to be a better guide than symptom reporting in selection of patients for urgent investigation.^21^ Introducing such practice would have cost-saving implications (improving resource allocation) while minimising unnecessary tests and associated harms (e.g. iatrogenic complication). This rationale is supported by a recent survey of 109 ACPGBI members; 81.3% believe the use of FIT has helped prioritise investigations.^8^ However, there will always remain a role for nonurgent colonoscopy in the diagnosis of other significant pathology.

The impact of single FIT in our centre has been highlighted; however, questions remain as to whether repeat FIT can aid decision making and reduce the false negative rate in the symptomatic population. There is a lack of evidence to support or refute the benefit of repeat FIT and how it could help in managing the endoscopy resource.^9,22^ A large retrospective evaluation of 28,622 patients by Hunt *et al* suggested that repeat FIT is a superior strategy compared with single FIT when managing symptomatic patients in primary care, without urgent referral.^23^ Prospective clinical studies are needed to determine whether more than one additional FIT is of benefit, and how FIT correlates with nonmalignant pathology.

We acknowledge the limitations of this study. The retrospective nature relies on accurate data collection at the time of referral. Our data give a snapshot of the experience of a single centre and therefore when interpreting more widely, consideration must be given to population factors such as deprivation indices and demographics. The timeframes assessed highlight uneven cohorts. Nonetheless, these periods were selected with the aim of assessing impact following both incorporation of FIT into clinical practice locally, and evolving NICE guidance.

The CRC TWW pathways of neighbouring secondary care providers may determine the referral practice of general practitioners in the region; this may have contributed to the increase in referrals to our service. A referral that meets NICE criteria will be placed on the TWW pathway in our centre irrespective of FIT result. However, this practice is not necessarily reflective of other centres. Consequently, an uneven distribution of referrals may occur.

## Conclusion

No significant change in CRC diagnosis occurred despite increasing TWW referral numbers. The utilisation of FIT during this timeframe (which has included periods of endoscopy rationing) has helped maintain the CRC detection rate while avoiding delay in diagnosis. Referrals from primary care remain above prepandemic levels, and services such as colonoscopy are consequently under ever-increasing strain.

The increasing use of FIT in primary care, without a parallel rise in TWW referral volume, indicates the growing importance of this investigation. The creation of national FIT guidance by ACPGBI and BSG may be the turning point that is required to help support both decision making in primary care and the prioritisation of secondary care resources. Our data demonstrate that FIT is already influencing investigation, but could be utilised further to reduce the burden on endoscopy, and support the recommendation that FIT-negative patients be managed in primary care. Due to the limited evidence base, questions remain regarding the most appropriate way of investigating this cohort. Additional low-cost strategies, such as repeat FIT in the symptomatic cohort, require evaluation in order to determine if they can provide further prioritisation or reassurance.
